# Comparison of Fucoidans from *Saccharina latissima* Regarding Age-Related Macular Degeneration Relevant Pathomechanisms in Retinal Pigment Epithelium

**DOI:** 10.3390/ijms24097939

**Published:** 2023-04-27

**Authors:** Philipp Dörschmann, Tabea Thalenhorst, Charlotte Seeba, Marie-Theres Tischhöfer, Sandesh Neupane, Johann Roider, Susanne Alban, Alexa Klettner

**Affiliations:** 1 Department of Ophthalmology, University Medical Center, University of Kiel, Arnold-Heller-Str. 3, Haus 25, 24105 Kiel, Germanyalexa.klettner@uksh.de (A.K.); 2 Pharmaceutical Institute, University of Kiel, Gutenbergstr. 76, 24118 Kiel, Germany; 3 Wyatt Technology Europe GmbH, Hochstrasse 12a, 56307 Dernbach, Germany

**Keywords:** sulfated fucan, retinal pigment epithelium-specific 65 kDa protein (RPE65), vascular endothelial growth factor (VEGF), phagocytosis, gene expression, protectin (CD59), trans-epithelial electrical resistance (TEER), interleukin, toll-like receptor, polyinosinic:polycytidylic acid (PIC)

## Abstract

Fucoidans from brown algae are described as anti-inflammatory, antioxidative, and antiangiogenic. We tested two *Saccharina latissima* fucoidans (SL-FRO and SL-NOR) regarding their potential biological effects against age-related macular degeneration (AMD). Primary porcine retinal pigment epithelium (RPE), human RPE cell line ARPE-19, and human uveal melanoma cell line OMM-1 were used. Cell survival was assessed in tetrazolium assay (MTT). Oxidative stress assays were induced with erastin or H_2_O_2_. Supernatants were harvested to assess secreted vascular endothelial growth factor A (VEGF-A) in ELISA. Barrier function was assessed by measurement of trans-epithelial electrical resistance (TEER). Protectin (CD59) and retinal pigment epithelium-specific 65 kDa protein (RPE65) were evaluated in western blot. Polymorphonuclear elastase and complement inhibition assays were performed. Phagocytosis of photoreceptor outer segments was tested in a fluorescence assay. Secretion and expression of proinflammatory cytokines were assessed with ELISA and real-time PCR. Fucoidans were chemically analyzed. Neither toxic nor antioxidative effects were detected in ARPE-19 or OMM-1. Interleukin 8 gene expression was slightly reduced by SL-NOR but induced by SL-FRO in RPE. VEGF secretion was reduced in ARPE-19 by SL-FRO and in RPE by both fucoidans. Polyinosinic:polycytidylic acid induced interleukin 6 and interleukin 8 secretion was reduced by both fucoidans in RPE. CD59 expression was positively influenced by fucoidans, and they exhibited a complement and elastase inhibitory effect in cell-free assay. RPE65 expression was reduced by SL-NOR in RPE. Barrier function of RPE was transiently reduced. Phagocytosis ability was slightly reduced by both fucoidans in primary RPE but not in ARPE-19. Fucoidans from *Saccharina latissima*, especially SL-FRO, are promising agents against AMD, as they reduce angiogenic cytokines and show anti-inflammatory and complement inhibiting properties; however, potential effects on gene expression and RPE functions need to be considered for further research.

## 1. Introduction

*Saccharina latissima* (SL), also known as sugar kelp or kombu, is a brown algae species from the family Laminariaceae. It grows along the coast of North America and Europe. Together with other *Laminaria* species, it forms kelp forests in the sublittoral zone below the low-water line. It is already widely used as food source and has a high-protein and low-fat content. As brown seaweed, it contains fucoidans, also known as sulfated fucans. Fucoidans are a group of sulfated polysaccharides which form complexes with alginates and cellulose in the cell wall of the algae, contributing to the robustness and protection against dehydration and environmental factors such as tides and water composition [1]. Fucoidans are a very heterogeneous group of biomolecules displaying different bioactivities depending on the origin, extraction, and purification, as these factors influence the final structure and composition of the fucoidan [2]. Many of these activities can be beneficial for humans. For example, they can have anti-inflammatory, anticoagulative, or antitumorigenic effects [3,4,5]. However, these effects may also depend on the model system applied. For example, the same fucoidan showed antiangiogenic effects in primary porcine pigment epithelium (RPE) and proangiogenic effects in human uveal melanoma cell lines [6,7]. Because of their high promising biological activities, fucoidans may be considered for a possible treatment option for retinal diseases like age-related macular degeneration (AMD) [8,9,10].

AMD is the main cause for severe vision loss and blindness of the elderly in industrialized nations, and the number of affected patients is expected to increase to 288 million by 2040 [11,12]. Early AMD phases are symptomless but may develop into symptomatic late forms. In the late atrophic (“dry”) form, RPE degenerates, followed by photoreceptors, which leads to retinal geographical atrophies. In the exudative (“wet”) form, excessive vascular endothelial growth factor (VEGF) secretion leads to uncontrolled vessel growth under and into the retina, leading to edema and, in severe cases, to bleeding or rupture of the retina [13]. Only exudative AMD is currently treatable, with anti-VEGF therapeutics, which have to be applied regularly into the eye [14]. The initial visual benefit of treatment is often lost in the long term. Indeed, VEGF antagonists may interfere with the physiological function of VEGF, as it is important for the fenestration of the blood vessels and confers cell protection [15]. Treatment options that can be applied at an earlier stage and may prevent the progression of AMD are highly warranted.

As a multifactorial disease, the main cause of AMD is still unknown, but pathological mechanisms like angiogenesis, inflammation, and oxidative stress clearly contribute to its development, interacting with each other [16,17,18]. In AMD development, the functions and interactions of the RPE, Bruch’s membrane, choroid, and retina are disturbed, with the RPE being the key player [19]. The RPE has manifold activities to maintain the proper functions of the metabolically highly active photoreceptors [20]. It builds a monolayer of tightly connected hexagonal cells forming tight junctions and the outer blood retinal barrier [20]. It secrets growth factors like VEGF and pigment epithelium-derived factor (PEDF) [20]. It has developed a plethora of antioxidative mechanisms due to the high metabolic activity and oxygen tension in the retina [21]. RPE phagocytes photoreceptor outer segments (POS) and recycles the visual pigment by enzymes such as retinal pigment epithelium-specific 65 kDa protein (RPE65). It also interferes with inflammatory processes, as it down regulates complement factors, forms the outer blood-retina barrier, and interacts with microglia [20,22]. CD59, also called protectin, is expressed by RPE cells, which contributes to the down regulation of the complement system by inhibiting the membrane attack complex (MAC) [23]. MAC activity correlates with AMD development [24]. The RPE expresses toll-like receptors (TLR) on its surface, which act as pattern recognition receptors making them able to detect danger-associated molecular patterns and pathogens [25,26]. Stimulating RPE cells with proinflammatory agents like lipopolysaccharide (LPS), polyinosinic:polycytidylic acid (PIC), tumor necrosis factor alpha (TNF), or Pam2CSK4 (Pam) can activate the secretion of proinflammatory cytokines and can influence RPE cellular functions such as barrier formation [22,27].

In previous studies a variety of fucoidans from different species were already tested concerning AMD relevant biological activity. The dependency of the biological activities on molecular weight, species, extraction method, purity, and cellular model systems was shown [28,29,30,31,32]. Even different batches from the same source material displayed heterogeneity [33]. In previous studies, fucoidans from SL showed promising results [29,31]. One of them (SL-Fuc) was from Faroe Islands, extracted with hot-water and CaCl_2_, and dialyzed prior to experimentation. It showed no relevant cell proliferation changing effects on tumor and healthy cells (short and mid-term) [28]. Furthermore, it could effectively inhibit VEGF secretion in human immortal adult RPE cell line ARPE-19 and showed VEGF binding affinity which was stronger than heparin [29]. It also displayed protective effects against oxidative stress induced by H_2_O_2_ in human uveal melanoma cell line OMM-1 [29]. It was remarkable that from five tested brown algae species it was the only fucoidan that was also protective against *tert*-butyl hydroperoxide induced oxidative stress in ARPE-19 and that lowered VEGF secretion in primary RPE [29]. In addition, SL fucoidans from Iceland which were enzymatically extracted, precipitated with CaCl_2_ and purified with ion-exchange chromatography (IEX), were tested [31]. Compared to other SL extracts, the IEX fraction 2 (SL-F2) had the most promising effects by showing protective effects against H_2_O_2_ in OMM-1 and lowering VEGF secretion in ARPE-19 by more than 50% [31].

In this study two SL fucoidans, one from the Faroe Islands (Denmark) and one from Norway, were tested concerning beneficial effects against pathologic mechanisms of AMD and their potential effect on RPE functions under inflammatory conditions. Fucoidans were examined regarding cell viability, protection against oxidative stress, VEGF secretion, interleukin 6 (IL-6) and interleukin 8 (IL-8) secretion and gene expression, barrier function, phagocytosis ability, and CD59 and RPE65 protein expression.

## 2. Results

### 2.1. Chemical Characterization

The two fucoidans investigated in this study were extracted from *Saccharina latissima* (SL) and purified as previously described [29,34,35]. Due to their extraction under non-degrading conditions, they have very high molecular weights. As typical for fucoidans from SL and other Laminariales, both SL-FRO and SL-NOR contained about 10% protein, tightly associated with the sulfated polysaccharide (glycoproteins or proteoglycans), whereas their total phenolic content (TPC) was very low (Table 1) [35,36]. However, the different origin and harvest time led to some pronounced differences between the two fucoidans (Table 1): (1) SL-FRO with a weight average molecular weight (*M_w_*) of 997 kDa was three times larger than SL-NOR (312 kDa); (2) the fucose content of SL-FRO (81 mol%) was significantly higher than that of SL-NOR (74 mol %); and (3) the degree of sulfation (DS) of SL-FRO (0.50) was consequently higher than that of SL-NOR (0.40). It should be mentioned that the fucose units were shown to be the sulfated monosaccharides in fucoidans [35].

### 2.2. Oxidative Stress Assays

**Figure 1:**
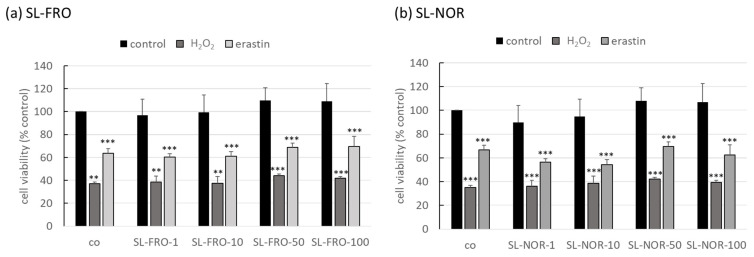
Oxidative stress assay with OMM-1. Human uveal melanoma cell line OMM-1 was treated with 1, 10, 50 or 100 µg/mL SL-FRO or SL-NOR and/or 500 µM H_2_O_2_ or 25 µM erastin at subconfluence for four hours. After that, cell viability was assessed with tetrazolium bromide assay (MTT). Absorption values were calculated relatively to the untreated control, set to 100%. Data were normally distributed (Shapiro–Wilk test) and one-sample *t*-test was conducted. ** *p* ≤ 0.01, *** *p* ≤ 0.001 (compared to co); co = control, n = 4 (number of independent experiments).

**Figure 2:**
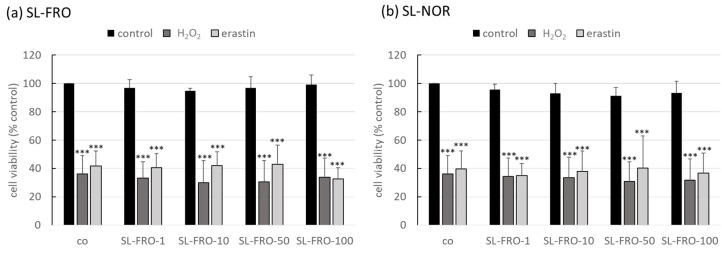
Oxidative stress assay with ARPE-19. Human retinal pigment epithelium cell line ARPE-19 was treated with 1, 10, 50, or 100 µg/mL SL-FRO or SL-NOR and/or 250 µM H_2_O_2_ or 20 µM erastin at subconfluence for 24 h. After that, cell viability was assessed with a tetrazolium bromide assay (MTT). Absorption values were calculated relatively to the untreated control, set to 100%. Data were normally distributed (Shapiro–Wilk test) and one-sample *t*-test was conducted. *** *p* ≤ 0.001 (compared to co); co = control, n = 4 (number of independent experiments).

Oxidative stress is one of the major pathological factors of AMD, and photoreceptors and RPE are exposed to a high amount of oxygenic radicals [21]. We used our established oxidative stress models, OMM-1 (Figure 1) and ARPE-19 (Figure 2) [29,37]. They were treated with 1, 10, 50, or 100 µg/mL fucoidans (SL-FRO or SL-NOR) and/or 250/500 µM H_2_O_2_ or 20/25 µM erastin for four/24 h, respectively. Cell viability was measured with tetrazolium bromide assay [3-(4,5-dimethylthiazol-2-yl)-2,5-diphenyltetrazolium bromide, MTT]. Data were calculated relative to untreated control, set to 100%. Fucoidans displayed a slightly stimulating effect in the higher concentrations, but their effect did not reach statistical significance. Overall, they showed no antiproliferative effects. Concerning OMM-1, viability was successfully decreased to 37 ± 2% or 35 ± 2% with 500 µM H_2_O_2_ (*p* = 0.0011/0.0001) and to 64 ± 4% or 67 ± 2% with 25 µM erastin (both *p* = 0.0001), both after 24 h of stimulation. Cell viability was only slightly increased by fucoidans after oxidative stress insult, with 50 µg/mL fucoidan showing the best effect, but this condition did not reach statistical significance. Concerning ARPE-19, cell viability was reduced by 250 µM H_2_O_2_ and 20 µM erastin with 36 ± 13% (both H_2_O_2_ treatments, *p* = 0.0002) and 42 ± 11 or 40 ± 13% (erastin, *p* = 0.0001), respectively. Again, fucoidans did not display any relevant or statistically significant protective effects.

### 2.3. Vascular Endothelial Growth Factor Secretion

**Figure 3:**
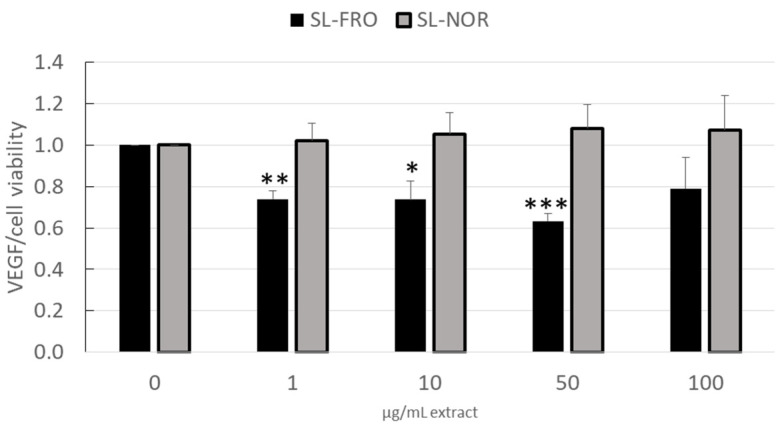
VEGF secretion in ARPE-19. Human retinal pigment epithelium cell line ARPE-19 was treated with 1, 10, 50, and 100 µg/mL SL-FRO or SL-NOR at confluence for three days. Tetrazolium bromide assay (MTT) was conducted to assess cell viability. Supernatant were collected for 24 h and investigated with ELISA for secreted vascular endothelial growth factor A (VEGF-A). VEGF in pg/mL was calculated in % to control and was normalized with cell viability data in % control. Data was normally distributed (Shapiro–Wilk test) and one-sample *t*-test was conducted. * *p* ≤ 0.05, ** *p* ≤ 0.01, *** *p* ≤ 0.001 (compared to 0 µg/mL fucoidan), n = 4 (number of independent experiments).

**Figure 4:**
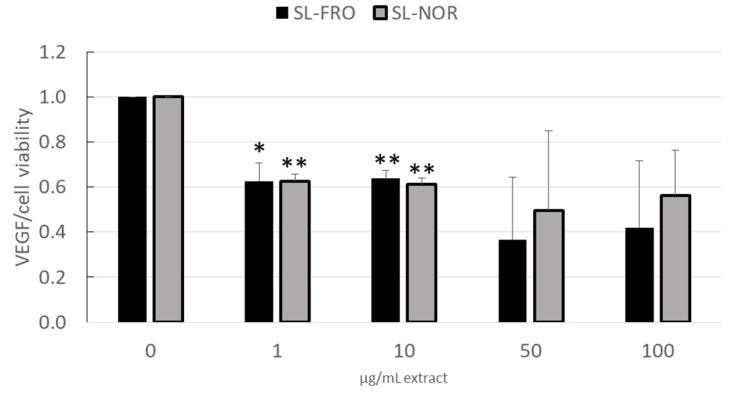
VEGF secretion in RPE. Primary porcine retinal pigment epithelium cells (RPE) were treated with 1, 10, 50, and 100 µg/mL SL-FRO or SL-NOR at confluence for seven days. Tetrazolium bromide assay (MTT) was conducted to assess cell viability. Supernatants were collected for four hours and investigated with ELISA for secreted vascular endothelial growth factor A (VEGF-A). VEGF in pg/mL was calculated in % to control and was normalized with cell viability data in % control. Data was normally distributed (Shapiro–Wilk test) and one-sample *t*-test was conducted. * *p* ≤ 0.05, ** *p* ≤ 0.01 (compared to 0 µg/mL fucoidan), n = 3 (number of independent experiments).

RPE cells secret high levels of VEGF, as VEGF is important for the fenestration of choroidal endothelial cells, but excessive secretion of VEGF strongly contributes to wet AMD [13]. We investigated the effects of 1–100 µg/mL fucoidan on VEGF secretion in ARPE-19 (three days stimulation, Figure 3) and RPE (seven days stimulation, Figure 4). Supernatants were collected for 24 h for ARPE-19 and for 4 h for RPE and applied in ELISA to test secreted VEGF-A. Cell viability was assessed with MTT assay and was used to normalize VEGF secretion.

Concerning ARPE-19, cell viability was not influenced after three days and remained stable around 100%. SL-NOR did not reduce VEGF secretion (Figure 3) at any tested condition in contrast to SL-FRO, which reduced VEGF in all tested concentrations with 1, 10, and 50 µg/mL fucoidan reaching statistical significance. It was reduced to 0.740 ± 0.040 [arb. unit], 0.737 ± 0.089 [arb. unit] and 0.630 ± 0.035 [arb. unit], respectively (*p* = 0.0015/0.0144/0.0004).

In primary RPE, cell viability was slightly reduced by 100 µg/mL SL-FRO to 82 ± 4% (*p* = 0.0230); for all other concentrations, it remained stable around 100%. VEGF secretion (Figure 4) was reduced by both fucoidans in all concentrations. A dose of 50 and 100 µg/mL showed lowest VEGF amount but had a higher deviation, therefore reaching no statistical significance. SL-FRO reduced VEGF to 0.626 ± 0.080 [arb. unit] at 1 µg/mL (*p* = 0.0219) and to 0.637 ± 0.036 [arb. unit] at 10 µg/mL (*p* = 0.0049). SL-NOR reduced VEGF to 0.626 ± 0.033 [arb. unit] at 1 µg/mL (*p* = 0.0037) and to 0.612 ± 0.030 [arb. unit] at 10 µg/mL (*p* = 0.0029).

### 2.4. Interleukin Secretion

**Figure 5:**
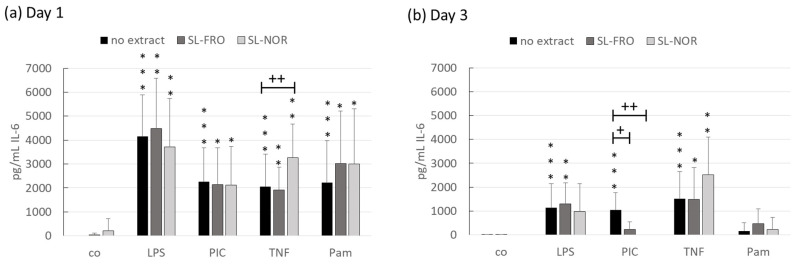
Interleukin 6 secretion of RPE. Primary porcine retinal pigment epithelium cells (RPE) were treated at confluence with 50 µg/mL SL-FRO or SL-NOR with and without 1 µg/mL lipopolysaccharide (LPS), 10 µg/mL polyinosinic:polycytidylic acid (PIC), 50 ng/mL tumor necrosis factor alpha (TNF), or 10 ng/mL Pam2CSK4 (Pam) for one (**a**) and three days (**b**). Supernatants were collected for 24 h and applied in ELISA for interleukin 6 (IL-6) measurement in pg/mL secreted protein. Data was normally distributed (Shapiro–Wilk test) and ANOVA with Dunnett’s test was used to evaluate significances. * *p* ≤ 0.05, ** *p* ≤ 0.01, *** *p* ≤ 0.001 (compared to co); + *p* ≤ 0.05, ++ *p* ≤ 0.01 (compared to stress control); co = control, n = 7 (number of independent experiments).

**Figure 6:**
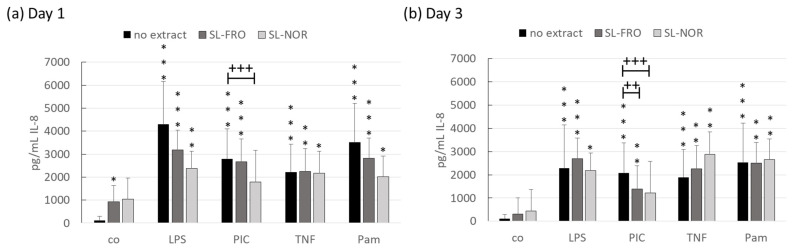
Interleukin 8 secretion of RPE. Primary porcine retinal pigment epithelium cells (RPE) were treated at confluence with 50 µg/mL SL-FRO or SL-NOR with and without 1 µg/mL lipopolysaccharide (LPS), 10 µg/mL polyinosinic:polycytidylic acid (PIC), 50 ng/mL tumor necrosis factor alpha (TNF), or 10 ng/mL Pam2CSK4 (Pam) for one (**a**) and three days (**b**). Supernatants were collected for 24 h and applied in ELISA for interleukin 8 (IL-8) measurement in pg/mL secreted protein. Data was normally distributed (Shapiro–Wilk test) and ANOVA with Dunnett’s test was used to evaluate significances. * *p* ≤ 0.05, ** *p* ≤ 0.01, *** *p* ≤ 0.001 (compared to co); ++ *p* ≤ 0.01, +++ *p* ≤ 0.001 (compared to stress control); co = control, n = 7 (number of independent experiments).

Fucoidan SL-FRO and SL-NOR (50 µg/mL) were tested for their anti-inflammatory properties. RPE were treated with fucoidans for one and three days with and without 1 µg/mL LPS, 10 µg/mL PIC, 50 ng/mL TNF, or 10 ng/mL Pam. Supernatants were collected for 24 h and evaluated in IL-6 (Figure 5) and IL-8 (Figure 6) ELISA. The effects of the fucoidans are clearly depending on the used proinflammatory agent so a general interleukin scavenging effect can be excluded.

Regarding IL-6 secretion, all used proinflammatory agents were able to activate RPE cells to secrete IL-6 (with exception of Pam after three days), while neither of the tested fucoidans activated IL-6 secretion. LPS lost this proinflammatory effect after three days when applied together with SL-NOR. Conversely, SL-NOR increased IL-6 secretion together with TNF compared to TNF alone with 3265 ± 1401 pg/mL compared to 2060 ± 1346 pg/mL (*p* = 0.0077). After three days of stimulation both fucoidans reduced PIC induced IL-6 secretion. SL-FRO reduced it to 216 ± 331 pg/mL (*p* = 0.0250) and SL-NOR completely diminished it to 0 ± 0 pg/mL (*p* = 0.0030).

Concerning IL-8 secretion, both fucoidans slightly increased cytokine secretion, but this effect was diminished after three days. In general, all proinflammatory agents activated IL-8 secretion for both tested time points. After one day of stimulation, both fucoidans tended to decrease LPS, PIC, and Pam-induced IL-8 secretion, whereas SL-NOR significantly decreased PIC-induced IL-8 secretion to 1791 ± 1371 pg/mL compared to 2792 ± 1302 pg/mL (*p* = 0.0415). After three days of stimulation, SL-FRO and SL-NOR significantly reduced PIC-induced IL-8 secretion to 1388 ± 1065 pg/mL and 1208 pg/mL, respectively, compared to PIC control with 2080 ± 1224 pg/mL (*p* = 0.0020/0.0004).

### 2.5. Interleukin Gene Expression

In addition to cytokine secretion, we investigated the effect of fucoidans on cytokine gene expression after PIC stimulation. Confluent primary porcine RPE was treated with 50 µg/mL SL-FRO or SL-NOR alone or together with 10 µg/mL PIC for three or seven days. Quantitative porcine gene expression of IL-6 (*IL6*) and IL-8 (*CXCL8 or IL8*) was assessed. Gene expression for glyceraldehyde-3-phosphate dehydrogenase (*GAPDH*) was used as endogenous control for normalization. Relative fold gene expression level RQ (=2^−ΔΔCT^), minimal RQ (RQ Min), maximal RQ (RQ Max), and *p*-value (student’s *t*-test) against untreated control or PIC control were evaluated with Thermo Fisher Connect (three days: Table 2; seven days: Table 3).

Three days of stimulation: Compared to control, PIC increased *IL8* expression up to 2.302 but reached no significance. For nearly all conditions IL6 expression tended to be decreased. SL-NOR significantly decreased *IL8* expression down to 0.238 (*p* = 0.0280). In addition, compared to PIC SL-NOR reduced *IL6* expression. Taken together, after three days of stimulation SL-FRO rather activates interleukins expression whereas SL-NOR reduces it.

Seven days of stimulation: Compared to control, PIC increased *IL8* expression slightly but not significantly. SL-FRO significantly increased *IL8* expression up to 50.009 (*p* = 0.0070). This effect was even stronger by dual stimulation with PIC with an RQ of 120.378 (*p* = 0.0100). Compared to PIC control, SL-FRO displayed a stronger *IL8* activation with RQ = 13.500 (*p* = 0.0210) on its own. Together with PIC it increased *IL8* expression up to 32.496 (*p* = 0.0090). Taken together, while PIC increases interleukin secretion, its effect on gene expression is less pronounced. Obviously, the effect of the fucoidans on cytokine secretion is not mirrored by their effect on gene expression.

### 2.6. Elastase Inhibition and Complement Inhibition

Typical structure-dependent activities of fucoidans include polymorphonuclear (PMN) elastase inhibition and complement inhibition [35]. Both contribute to their anti-inflammatory potency and are, therefore, of interest concerning the interference of fucoidans with the AMD pathogenesis. Notably, the complement inhibition is currently considered as the top strategy for the development of novel AMD therapies [38,39,40]. Both activities are suitable parameters for an AMD screening of test compounds.

**Figure 7:**
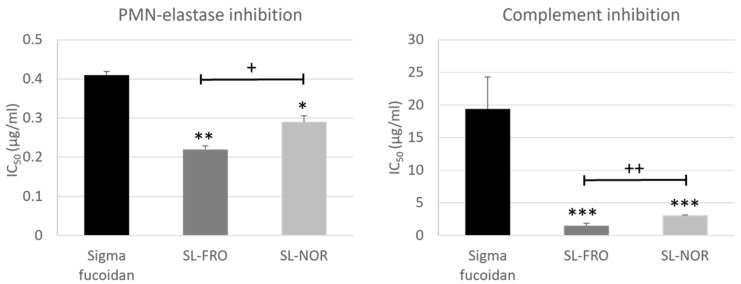
IC_50_ (µg/mL) of the fucoidans for polymorphonuclear (PMN) elastase inhibition and complement inhibition. Data was normally distributed (Shapiro–Wilk test) and ANOVA with Dunnett’s test was used to evaluate significances. * *p* ≤ 0.05, ** *p* ≤ 0.01, *** *p* ≤ 0.001 (compared to Sigma fucoidan); + *p* ≤ 0.05, ++ *p* ≤ 0.01 (between SL-FRO and SL-NOR); IC_50_ = half maximal inhibitory concentration, n ≥ 2 (number of analysis).

Both SL-FRO and SL-NOR turned out to be superior to the widely used commercially available Sigma-Aldrich *Fucus vesiculosus* fucoidan (Sigma fucoidan [6,7,28,29,37]) (Figure 7). In the elastase assay, the half maximal inhibitory concentration (IC_50_) of SL-FRO (IC_50_ 0.22 µg/mL, *p* = 0.0022) and SL-NOR (IC_50_ 0.29 µg/mL, *p*= 0.0121) were significantly lower than Sigma fucoidan (IC_50_ 0.41 µg/mL). In addition, SL-FRO was significantly more active in elastase inhibition than SL-NOR (*p* = 0.0272). Their IC_50_ in the complement assay (SL-FRO: 1.5 µg/mL, *p* = 0.0000; SL-NOR: 3.1 µg/mL, *p* = 0.0001) were one order of magnitude smaller than that of Sigma fucoidan (19.4 µg/mL) and SL-FRO with its higher *M_w_* and higher DS was significantly more active than SL-NOR (*p* = 0.0045). Thus, these two fucoidans are very promising concerning early complement inhibition.

**Figure 8:**
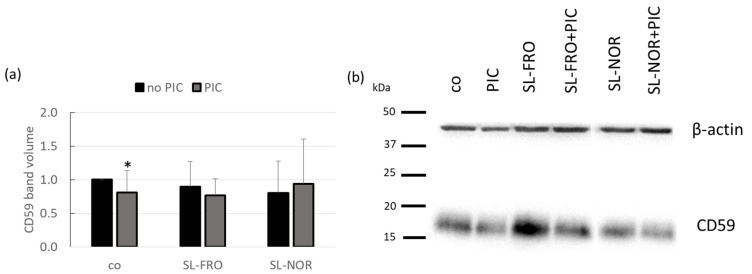
CD59 protein expression of RPE. Primary porcine retinal pigment epithelium (RPE) cells were treated with 50 µg/mL SL-FRO or SL-NOR with and without 10 µg/mL polyinosinic:polycytidylic acid (PIC) for three days. Cell lysates were applied in western blot to assess CD59 (protectin, 18 kDa) and β-actin (48 kDa) expression. An exemplary blot is shown (**b**). Band volumes were evaluated with TotalLab. CD59 volumes were normalized with data of β-actin. Data was calculated relative to control (set to 1.0, (**a**)). Data was normally distributed (Shapiro–Wilk test). Significances were evaluated with one-sample *t*-test. * *p* ≤ 0.05 (compared to co); co = control, n = 5 (number of independent experiments).

After testing the overall complement inhibition, we assessed the influence on the important complement regulator CD59, which is expressed by RPE [23]. Protein expression of CD59 (Figure 8) in primary porcine RPE was determined by western blotting. Cells were treated with 50 µg/mL SL-FRO or SL-NOR with and without 10 µg/mL PIC for three days. Band volumes CD59 were normalized with data of β-actin. Data was set in relation to untreated control (set to 1.0). Densitometric evaluation showed that PIC stimulation reduced CD59 expression to 0.811 ± 0.321 (*p* = 0.0460). The reduced expression of CD59 under PIC stimulation was lost after additional stimulation with either fucoidan. Qualitative assessment indicated that SL-FRO tends to exhibit stronger CD59 expression than control as demonstrated in Figure 8b.

### 2.7. Barrier Measurement

**Figure 9:**
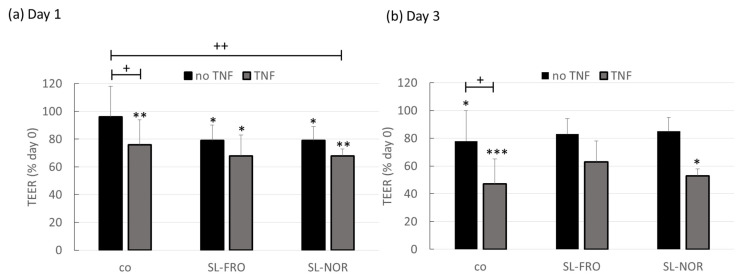
Barrier function of RPE. Primary porcine pigment epithelium (RPE) cells were treated with 50 µg/mL SL-FRO or SL-NOR and/or 50 ng/mL tumor necrosis factor α (TNF) in 12-transwell plates. Transepithelial electrical resistance (TEER) was measured in Ω*cm^2^ before stimulation as well as after one and three days. TEER was set in relation to the day 0 values and are depictured in %. Data was normally distributed and one-sample *t*-test was conducted. * *p* ≤ 0.05, ** *p* ≤ 0.01, *** *p* ≤ 0.001 (compared to day 0); + *p* ≤ 0.05, ++ *p* ≤ 0.01 (compared to control); n = 4 (number of independent experiments).

Primary porcine RPE cells were seeded on 12-transwell plates. Transepithelial electrical resistance (TEER) was measured at day 0 (before treatment) immediately before the cells were treated with 50 µg/mL SL-FRO or SL-NOR and/or 50 ng/mL TNF. TNF was chosen, as we previously have shown that TNF reduces barrier function in primary RPE [22]. TEER was measured again after one and three days. TEER was calculated in relation to the corresponding day 0 values, set to 100% (Figure 9).

After one day, cells treated with fucoidans or/and TNF showed a reduced cell barrier compared to day 0, reaching TEER of 68% if fucoidan and TNF are applied together. However, only TNF and dual stimulation of SL-NOR and TNF were significantly lower than control, with 76 ± 18% (*p* = 0.0142) and 68 ± 5% (*p* = 0.0026), respectively. After three days control, TNF and dual stimulation of TNF and SL-NOR showed significant barrier reduction compared to day 0, whereas SL-FRO conditions regenerated. Compared to control, TNF lowered TEER to 47 ± 27% (*p* = 0.0134) at day 3. Taken together, TNF and fucoidans interfere with barrier functions of RPE, whereas fucoidans showed potential to reverse this effect after mid-term stimulation.

### 2.8. Protein Expression

**Figure 10:**
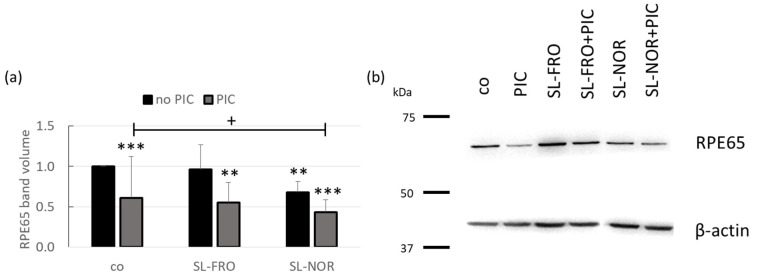
RPE65 protein expression of RPE. Primary porcine retinal pigment epithelium (RPE) cells were treated with 50 µg/mL SL-FRO or SL-NOR with and without 10 µg/mL polyinosinic:polycytidylic acid (PIC) for three days. Cell lysates were applied in western blot to assess RPE-specific 65 kDa protein (RPE65) and β-actin (48 kDa) expression. An exemplary blot is shown (**b**). Band volumes were evaluated with TotalLab. RPE65 volumes were normalized with data of β-actin. Data was calculated relative to control (set to 1.0, (**a**)). Data was normally distributed (Shapiro–Wilk test). Significances were evaluated with one-sample *t*-test. ** *p* ≤ 0.01, *** *p* ≤ 0.001 (compared to co); + *p* ≤ 0.05 (compared to PIC); co = control, n = 5 (number of independent experiments).

Protein expression of RPE65 (Figure 10) in primary porcine RPE was determined in western blotting. Cells were treated with 50 µg/mL SL-FRO or SL-NOR with and without 10 µg/mL PIC for three days. Band volumes were densitometrically evaluated. Band volumes of RPE65 were normalized with data of β-actin. Data were set in relation to untreated control, set to 1.0. Exemplary blots are depictured in Figure 10b.

Densitometric evaluation showed that PIC significantly reduced RPE65 expression to 0.610 ± 0.515 (*p* = 0.0010). In addition, SL-NOR significantly reduced RPE65 expression to 0.680 ± 0.133 (*p* = 0.0020), while SL-FRO did not influence RPE65 expression (1.000 ± 0.303, *p* = 0.814). Combinations of SL-FRO and SL-NOR with PIC did reduce RPE65 expression [0.554 ± 0.246 (*p* = 0.0070) and 0.434 ± 0.149 (*p* = 0.0002)], respectively. SL-NOR plus PIC condition was significantly lower than PIC alone (*p* = 0.0308).

### 2.9. Phagocytosis Assay

**Figure 11:**
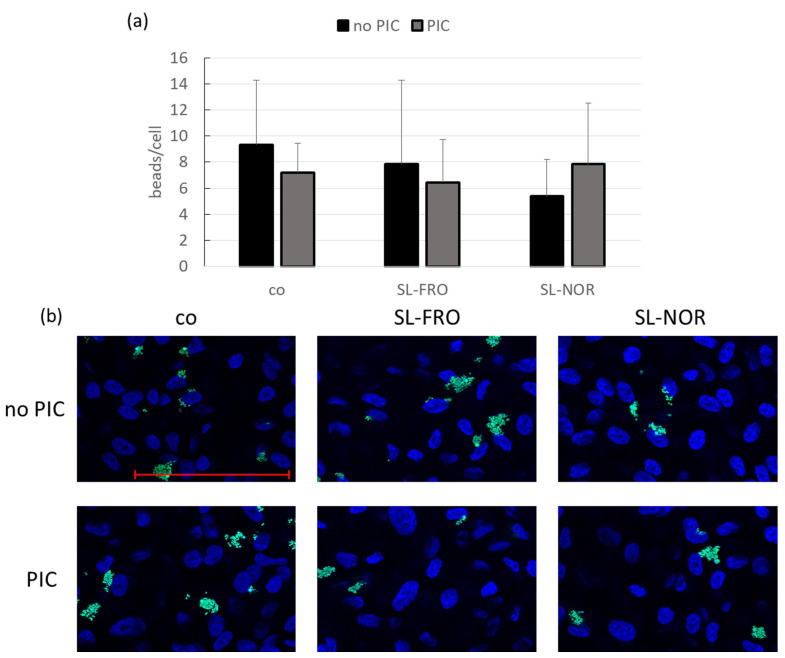
ARPE-19 phagocytosis. Human RPE cell line ARPE-19 was treated with 50 µg/mL SL-FRO or SL-NOR with and without 10 µg/mL polyinosinic:polycytidylic acid (PIC) for three days. To measure phagocytosis, fluorescent beads opsonized with photoreceptor outer segments were added to the cells (green dots, (**b**)). Cells were fixated with paraformaldehyde and treated with Fluoromount-G™ and 4′,6-diamidino-2-phenylindol (DAPI) to mark cell nuclei (blue circles, (**b**)). Photos were taken with an immunofluorescence microscope (640× magnification, red scale bar = 100 µm). Nuclei were counted manually and beads with Fiji Software (ImageJ2). Number of beads was put in relation to the cell nuclei (**a**). Data were normally distributed (Shapiro–Wilk test). Significances were determined with analysis of variance (ANOVA) and post-hoc Dunnett’s multiple comparison test. Significance was not reached for any treatment. co = control, n = 15 (number of independent experiments).

**Figure 12:**
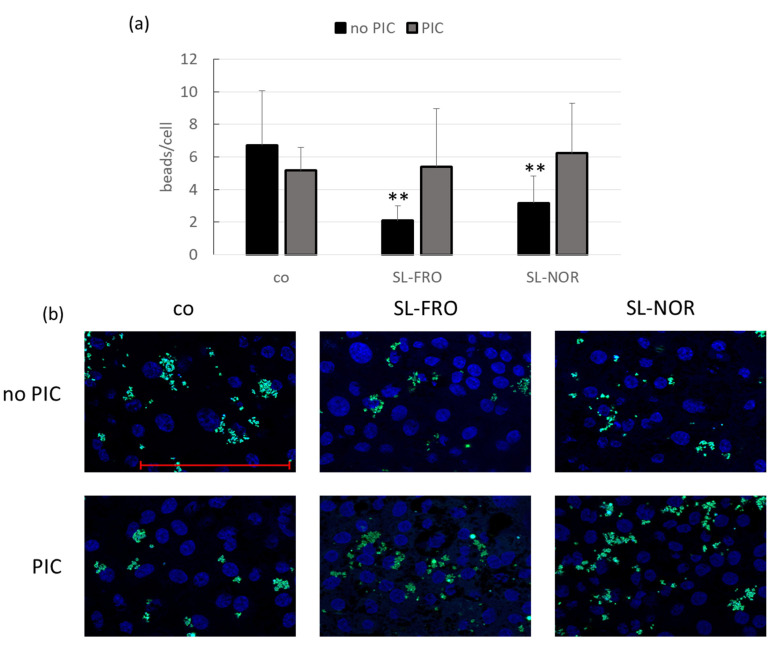
RPE phagocytosis. Primary porcine pigment epithelium (RPE) cells were treated with 50 µg/mL SL-FRO or SL-NOR with and without 10 µg/mL polyinosinic:polycytidylic acid (PIC) for three days. To measure phagocytosis, fluorescent beads opsonized with photoreceptor outer segments were added to the cells (green dots, (**b**)). Cells were fixated with paraformaldehyde and treated with Fluoromount-G™ and 4′,6-diamidino-2-phenylindol (DAPI) to mark cell nuclei (blue circles, (**b**)). Photos were taken with an immunofluorescence microscope (640× magnification, red scale bar = 100 µm). Nuclei were counted manually and beads with Fiji Software (ImageJ2). Number of beads was put in relation to the cell nuclei (**a**). Data were normally distributed (Shapiro–Wilk test). Significances were determined with analysis of variance (ANOVA) and post-hoc Dunnett’s multiple comparison test. ** *p* ≤ 0.01 (compared to co), co = control, n = 15 (number of independent experiments).

Primary porcine RPE and human RPE cell line ARPE-19, respectively, were treated with 50 µg/mL SL-FRO or SL-NOR with and without 10 µg/mL PIC for three days. A phagocytosis assay was conducted. Blue cell nuclei were counted per hand and green labeled beads per Fiji software. The beads number was set in relation to the cell nuclei as an estimate for cell number (ARPE-19: Figure 11 and RPE: Figure 12). For ARPE-19, no significant results were achieved (compared to control or PIC), but PIC and SL-NOR tended to decrease phagocytosis slightly. Tests were repeated with primary RPE. Here, all fucoidans and PIC lowered phagocytic uptake of beads with the fucoidans reaching significance with SL-FRO reducing it to 2 ± 1 beads/cell (*p* = 0.0020) and SL-NOR to 3 ± 2 (*p* = 0.0060). Of note, fucoidans and PIC nullified the phagocytosis reducing effects when applied together in comparison to their single treatments.

## 3. Discussion

Fucoidans are marine polysaccharides from brown algae cell walls which display a variety of promising biological activities throughout the literature, e.g., antioxidative, anticoagulant, and immunoregulatory effects [41]. But their high heterogeneity in composition, structure and origin, and their contradicting effects depending on the model systems, make it necessary to categorize them in order to choose the appropriate fucoidan for the desired purpose. Fucoidans are currently under investigation for their suitability as treatment and prevention for AMD [9,10].

The main cause for AMD is still unknown, but four pathological pathways highly contribute to the development of this disease. This factors are inflammation, oxidative stress, lipid accumulation, and, in case of wet AMD, angiogenesis [13,17,18]. Currently only wet AMD can be slowed down but not cured or prevented by anti-VEGF therapeutics, which need to be repeatedly injected into the vitreous [14]. A new agent that targets more of these pathological factors and may prevent progression is highly desired [10]. Several fucoidans were tested in previous studies, showing their promising effects in ocular cell systems and indicating correlation of factors like species, extraction method, and molecular weight with biological activities [9]. Fucoidans from brown algae species SL and *Laminaria hyperborea* as well as the commercially available *Fucus vesiculosus* fucoidan from Sigma-Aldrich displayed the most promising activities in antiangiogenic and antioxidative activities [7,29,30,31]. A common factor in all promising fucoidans was their high molecular weight. Indeed, low-molecular weight fucoidans have been described to rather act proangiogenic [42]. The high-molecular weight fucoidans from past studies were purely extracted with high content of fucose and a high degree of sulfation [30,31,37]. Moreover, we have previously shown that the most promising *Laminaria hyperborea* fucoidan does not interfere with important RPE activities and displayed anti-inflammatory properties [43]. This study focused on the other promising seaweed species, SL, with two higher molecular weight fucoidans from the coast of Norway (SL-NOR) and of the Faroe Islands (Denmark, SL-FRO). They were tested regarding antioxidative, antiangiogenic, anti-inflammatory, and complement inhibitory properties in ocular cell models and influence on porcine RPE cellular functions. Primary porcine RPE is a suitable model for adult human RPE [44]. The porcine ocular system is much closer than the eyes of mice to the human ocular system. Pigs show a similar visual behavior like diurnal activity, similar size and anatomy of the eye ball and lens, similar number and distribution of cones and rods, as well as similar gene and protein profile [44,45,46].

Concerning testing models of this study, we used well established primary porcine RPE, adult human ARPE-19, and uveal melanoma cell line OMM-1 [44,47]. The different models were chosen in order to model the different aspects of AMD pathology. For oxidative stress, OMM-1 and ARPE-19 cells were used, as primary RPE are highly resistant to oxidative stress and, therefore, not suitable [21,37]. Indeed, the ARPE-19 cell line is a suitable model for RPE cells [48], but differs from differentiated RPE cells, e.g., they do exhibit a different gene expression pattern, do not develop a good barrier function, and exhibit a less distinct morphology [44,49,50]. These aspects need to be considered when comparing the effects of fucoidan. Primary porcine RPE cells, on the other hand, are highly differentiated cells, which represent the in situ RPE cells more closely. They exhibit a highly differentiated phenotype, can reach high barrier function, and display typical RPE function, such as phagocytosis [44]. While they are not of human origin, they exhibit morphological and molecular features that resemble the human RPE more closely [44,50]. As they are derived from distinct individuals, the variability of the results is generally higher compared to results obtained with cell lines.

No antiproliferative were detected in any model until seven days of treatment, which was also confirmed with other SL fucoidans in past studies [28,31]. It has been described that fucoidans can interfere with the adherence of cells through interaction with extracellular membrane components (e.g., fibronectin) [51]. Therefore, in order to control for potential detachment of cells, VEGF measurements were normalized with MTT data.

OMM-1 were used as an appropriate model for oxidative stress insult because of their sensitivity and decreased superoxide dismutase expression [52]. RPE are intrinsically highly protective against oxidative stress, especially when differentiated and when containing melanin [21]; therefore, the human cell line ARPE-19 was used, which is more susceptible towards oxidative stress. In this study both tested SL fucoidans did not show protective effects against direct oxidative stress due to H_2_O_2_ or indirect oxidative stress by ferroptosis insult via erastin in either cell systems. This is in contrast to previous studies where we could show the protective effect of SL fucoidans against oxidative stress insult in ARPE-19 or OMM-1 (SL-Fuc and SL_F2 [29,31]). One of these fucoidans was also from the Faroe Islands (SL-Fuc) but had a molecular weight of 1401 kDa, which is larger [34] compared to the fucoidans of this study, which are below 1000 kDa. It was speculated that fucoidans are not reactive oxygen species (ROS) scavengers, but their anti-oxidative property may be rather due to co-extracted compounds like polyphenols [33,53]. Instead, fucoidans could interact with molecular signaling in the oxidative stress counteract pathway [37]. The fucoidans of this study do not show these activities. This may be related to the molecular weight, as the protective SL-Fuc was significantly larger [29], and it was shown that higher molecular weights are more promising for oxidative stress protection [30,31,37]. In addition, polyphenols might contribute to antioxidative effects, as the protective SL fucoidan from the former study showed a much higher polyphenol content with 9.7 µg GAE/mg (gallic acid equivalents) [35], compared to 1.5 and 3.7 µg GAE/mg from the fucoidans of this study.

Concerning effects on angiogenesis, we focused on the secretion of VEGF as the main factor in development of wet AMD and only target of treatment with current therapeutics at the time of this study [14,16]. Here it was shown that, depending on the concentration, VEGF secretion is remarkably reduced in ARPE-19 by SL-FRO and in primary RPE by both SL fucoidans, with 50 µg/mL being most effective. RPE VEGF secretion is reduced in a similar manner by both fucoidans, but SL-FRO seems to be more effective, as it also inhibits VEGF in ARPE-19. Of note, SL-FRO has a much higher molecular weight with 997 kDa than SL-NOR with 312 kDa, and the fucose content and degree of sulfation are higher. This strongly indicates that molecular weight is the most important factor determining the effect on VEGF secretion. The high-molecular weight fucoidan SL-Fuc (1401 kDa [34]) from a former study also inhibited VEGF in both ARPE-19 and RPE [28]. The chemical differences between different fucoidans are correlated to the time of harvest [36], indicating that time and place of algae harvest are important factors too.

Furthermore, in a former study the lack of VEGF receptor 1 (VEGFR-1 or FLT) gene expression in this ARPE-19 batch was shown [47]. VEGFR-1 works as co-factor and binding supporter for VEGF receptor 2 (VEGFR-2 or KDR) and enforces the binding and activity of VEGF to VEGFR-2, which then mediates cellular response [54]. In addition, ARPE-19 cells secret way less VEGF than primary RPE [29]. The molecular differences in these two cell systems may explain the different effects of the fucoidans. Due to the lack of VEGFR-1 in ARPE-19 and the smaller amount of VEGF, the inhibiting effect of SL-FRO may be mainly mediated by preventing VEGF binding to VEGFR-2 via antagonizing it or by blocking the receptor [55]. SL-NOR, however, shows only an effect in primary RPE that express VEGFR-1. Thus, SL-NOR may not interfere with VEGF and VEGFR-2 (like SL-FRO) but rather with the co-factors (like VEGFR-1 or heparan sulfate) supported binding of VEGF. This may be due to different molecular sizes.

For further experiments we focused on 50 µg/mL fucoidan, because it showed the highest effect in VEGF inhibition in ARPE-19 and RPE (this was also the case in a former study [30]). The decision was mainly made because VEGF inhibition testing was used as pre-screening method to choose appropriate concentrations and focus on one to reduce the amount of experiments. In our experience, lower concentrations may not be effective enough and 100 µg/mL could lead to toxic or effects or detachment of cells like shown for SL-FRO, which reduced cell viability of RPE slightly at 100 µg/mL. It seems that 100 µg/mL activates different pathways that interfere slightly with the primary VEGF reduction, explaining also the higher biological variance.

Further experiments tested anti-inflammatory effects as well as RPE cellular functions with and without inflammatory activation. LPS as activator for toll-like receptor 4 (TLR-4), PIC as activator for (TLR-3), Pam as activator of TLR-2/TLR-1, and TNF as cytokine for TNF receptor related pathways were all efficient in inducing proinflammatory cytokine release in the RPE [22,27]. Concentrations, times, and investigated interleukins were chosen based on these previous studies [23]. Cell viability was not changed under proinflammatory stimulation. We detected a small IL-8 induction after SL-FRO treatment, which was lost after three days. In addition, SL-NOR slightly increased TNF induced IL-6 secretion after one day. However, all fucoidans effectively reduced PIC induced IL-6 and IL-8 secretion. This indicates a potential interference with the TLR3- related inflammatory pathway. Of note, fucoidans from other brown algae species rather reduced the TLR-2 and TLR-4 pathways in other cell models, but in our case only a tendential reduction for LPS induced IL-8 secretion was seen after one day [56,57]. This dependency on fucoidan and model system has been described before [7,58]. In addition, it was described that low-molecular weight fucoidan counteract LPS induced reactions [59], and we used higher molecular weight fucoidans, which seem to be more efficient on the TLR-3 pathway. It has also to be considered that previous studies focused on macrophages [60], which behave differently from RPE [20], which may secrete cytokines also for regulatory interaction with monocytic and microglia cells [22,61]. TLR-3 is of high interest for AMD related research, as it is activated by degenerating RPE cells and may contribute with chronic inflammation process to this development [62]. In addition, TLR-3 can activate VEGF secretion [63]. Fucoidans could counteract both the inflammatory and angiogenic pathways of TLR-3, but the exact mode of action is unknown. Due to the effect of fucoidans on TLR-3-mediated cytokine secretion, we decided to focus on PIC as proinflammatory stimulus. We tested the *IL6* and *IL8* gene expression after stimulating with fucoidans and/or PIC. Surprisingly, PIC did not induce proinflammatory gene expression of the interleukins. Fucoidans, on the other hand, displayed effects on interleukin expressions. SL-NOR decreased *IL8* gene expression after three days, whereas SL-FRO increased it after seven days. The first corresponds well to the secretion of IL-8 showing in the cytokine release assay, while the latter shows contradicting effects as SL-FRO reduced IL-8 secretion but increased gene expression. IL-8 is also important as a proangiogenic factor [64], and maybe the antiangiogenic effects of SL-FRO interfered with the gene expression and proangiogenic activity of IL-8. In a recent study [65], LPS was a more suitable inductor for *IL6* and *IL8* gene expression. Further gene expression tests with these fucoidans after LPS insult are planned.

An important factor for AMD development is the complement system, and RPE shows also regulatory effects on the basolateral site [20]. CD59 is an important complement regulator and inhibitor of MAC [66], and CD59 reduction is associated with AMD development [67]. PIC could lower the expression of CD59 which indicates that TLR-3 pathways also interfere with the regulation of the complement pathway. Fucoidans counteracted the PIC reduced CD59 expression, indicating a protective effect on complement inhibition under inflammation. In terms of elastase and complement inhibition both fucoidans were more active than the reference substance and also SL-FRO showed a better activity than SL-NOR. These activity differences may be due to the fact that both biological effects were shown to be dependent on the *M_w_* and the DS of sulfated polysaccharides, whereby the complement inhibition is especially sensitive to the *M_w_* [68,69,70]. Our data indicate a complement inhibiting effect of fucoidans, which make them particularly interesting for AMD treatment.

Concerning barrier function, we tested TNF, as it was shown before that it can decrease the RPE barrier function [71]. Here we could show that the barrier indeed is reduced by TNF but also slightly reduced after fucoidan treatment. TNF and combination of TNF and SL-NOR reduced the barrier compared to control after three days. However, the barrier reducing effect of fucoidans was transient after which it normalized, which was not the case in TNF related treatment. Therefore, the barrier reduction may be of little biological relevance.

RPE65 is an important key enzyme in visual pigment recycling by regenerating 11-*cis*-retinal [72]. RPE65 expression was decreased by SL-NOR and PIC. TLR-3 activation seems to interfere with handling of visual pigment recycling. SL-NOR worsens this effect, which needs to be considered for further studies. However, it could also be interpreted in the sense of a protective effect. Di-retinal conjugate A2E as part of lipofuscin accumulates throughout phototransduction process from outer photoreceptor segments and, thereby, contributes to the dry form of AMD [73]. The reduction of the visual recycling could prevent this effect. Bavik et al. tested emixustat as an RPE65 inhibitor [74]. They were able to show that a three month treatment with emixustat reduced the generation of A2E in an animal model, which renders this agent promising for treatment and prevention of dry AMD [74], which could also be the case for the fucoidans from this study.

To ensure photoreceptor function, RPE has to phagocyte photoreceptor outer segments. We investigated the phagocytosis ability of RPE after fucoidan stimulation with and without inflammatory activation. In ARPE-19 no effect could be detected. It should be noted, however, that in our experience, ARPE-19 cells generally show a weaker phagocytosis activity than primary RPE. In primary RPE, PIC tendentially reduced phagocytosis ability. Of note, both fucoidans significantly reduced this activity when applied alone. Interestingly, however, if PIC and fucoidans are applied together this effect was canceled. The effect of PIC stimulation on RPE is consistent with a previous study in which rubella virus-infected human RPE showed a decreased phagocytosis activity [75]. As PIC basically imitates a viral infection, the decrease of phagocytosis under PIC is consistent.

With this study we could show the effectiveness of SL fucoidans from Norway and the Faroe Islands. They showed anti-inflammatory activities by inhibiting TLR3-related IL-6 and IL-8 secretion, or lowering PMS elastase activity. In addition, they showed antiangiogenic effects by reducing secreted VEGF in RPE. Moreover, both fucoidans effectively inhibit complement. The two SL fucoidans showed different activities regarding influence on RPE cellular functions. Concerning reduction of RPE65 expression and barrier function, SL-NOR may increase the effect of the stress stimulus, while SL-FRO effects were modest or showed no negative effect at all. Taken together, SL-FRO as a high-molecular weight fucoidan with its higher efficiency in VEGF inhibition and better results in RPE functional testing is recommend for further research concerning age-related macular degeneration.

## 4. Material and Methods

### 4.1. Algal Material and Extraction Method

The dried SL seaweed was kindly provided by Coastal Research & Management GmbH (Kiel, Germany). Names, harvest place, and harvest date are shown in Table 4. Fucoidans were extracted by the Pharmaceutical Institute Kiel as previously described [36]. For defatting of the pulverized seaweed, Soxhlet extraction with 99% (*v*/*v*) ethanol was conducted. The extraction was performed with 2% CaCl_2_ in water for two hours at 85 °C with reflux condition. Supernatant was evaporated and treated with cold 60% ethanol. Finally, the solution was centrifugated, dissolved in aqua dest. as well as dialyzed and lyophilized. Brown algae used in this study came from countries without access regulation to their genetic resources (i.e., Norway and the Faroe Islands).

### 4.2. Chemical Characterization

#### 4.2.1. Elemental Analysis

The content of carbon, hydrogen, nitrogen, and sulfur of the fucoidans were determined by elemental analysis as previously described [35] using a varion MICRO cube elemental analyzer (Elementar Analysensysteme GmbH, Langenselbold, Germany; calibrator: sulfanil amide). The degree of sulfation (DS: number of sulfate groups per monosaccharide) was calculated based on the sulfur content (%). Total protein content was estimated by multiplying the nitrogen content (%) by 6.25 [76].

#### 4.2.2. Monosaccharide Composition by Gas–Liquid Chromatography of Alditol Acetates

The neutral monosaccharide composition of the fucoidans was determined by converting the samples into alditol acetate derivatives as previously described [77,78], which were then analyzed by gas–liquid chromatography using a Agilent 7890B system (Agilent Technologies, Santa Clara, CA, USA) with FID (flame ionization detection) [32,53].

#### 4.2.3. Molecular Weight Determination by Size-Exclusion Chromatography with Multiple Detection

The average molecular weight (*M_w_*, Da) of the fucoidans was determined by size-exclusion chromatography coupled with multiple detection [79]. The separation system was an Agilent 1260 Infinity II standard LC system (Agilent Technologies, Santa Clara, CA, USA) with an OHpak SB-G guard column and two coupled OHpak-LB 806 M columns (Resonac Europe GmbH, Shodex Business, Muenchen, Germany). The detection system consisted of a UV detector (Agilent 1260 Infinity II VWD), a Wyatt DAWN^®^ HELEOS^®^ 8+ multiple angle light scattering (MALS) detector with an integrated Wyatt QELS dynamic light scattering (DLS) module, a ViscoStar^®^ on-line differential viscometer, and an Optilab^®^ T-rex differential refractometer (both Wyatt Technology Europe GmbH, Dembach, Germany) connected in series. The mobile phase was PBS buffer (50 mmol/L Na2HPO4–NaH2PO4, 150 mmol/L NaCl, pH 7.0) filtered through an 0.1 µm membrane filter. The samples were dissolved in the mobile phase (2 mg/mL) and filtered (0.45 µm) before injection (100 µL). The applied flow rate was 1 mL/min. The dn/dc value of 0.135 mL/g was determined by batch experiments using an Optilab^®^ T-rex differential refractometer (658 nm) [79].

#### 4.2.4. Total Phenolic Content

TPC of the fucoidans was determined by the Folin–Ciocalteau method with adapted volumes in a 96-well microplate (nunc™ 269620, Thermo Fisher Scientific, Waltham, MA, USA) as previously described [33,80]. Gallic acid (GA) and ascorbic acid were used as reference compounds. The TPC of the fucoidans was expressed as gallic acid equivalents (GAE) µg GAE/mg dry substance.

### 4.3. Fluorogenic Elastase Activity Assay

Elastase inhibitory activity of the fucoidans was examined by a fluorogenic microplate assay (nunc™ 237108, Thermo Fisher Scientific, Waltham, MA, USA) using elastase from human polymorph nuclear granulocytes (PMN) (art.-No. 324681; Merck Chemicals GmbH, Darmstadt, Germany) and the substrate I-1270 (MeOSuc-Ala-Ala-Pro-Val-7-amido-4-methylcoumarin; Bachem AG, Bubendorf, Switzerland). The assay was performed as previously described [81,82]. The concentrations of the fucoidans for 50% inhibition of elastase activity [IC_50_ (µg/mL)] were calculated via the concentration-dependent inhibition curves.

### 4.4. Hemolytic Classical Complement Modulation Assay

Complement inhibiting potency of the fucoidans was investigated by assessing their effect on the lysis of sheep erythrocytes induced by classical pathway activation (Hemolytic System for CFT, HS-023, Labor Dr. Merk und Kollegen GmbH, Ochsenhausen, Germany) as previously described [68]. As complement source 2.1% human pooled serum was used. The IC_50_ (µg/mL) of the fucoidans were calculated via the concentration dependent inhibition curves.

### 4.5. Cell Culture

Uveal melanoma cell line OMM-1 (RRID: CVCL_6939) [83] was kindly provided by Dr. Sarah Coupland. The cell line was seeded in 96-well plates (200,000 cells/mL) and treated at subconfluence 24 h after seeding. Media was RPMI 1640 (Pan-Biotech, Aidenbach, Germany) containing 1% penicillin/streptomycin (Sigma-Aldrich, St. Louis, MO, USA) and 10% fetal bovine serum (Thermo Fisher Scientific, Waltham, MA, USA). Cells were passaged 1:4 two times a week after reaching confluence.

Human RPE cell line ARPE-19 (ATCC, RRID: CVCL_0145) is an immortal cell line from an adult donor with age of 19 [48]. Cells were seeded in 96-well or 12-well plates (100,000 cells/mL) and treated after 24 h in 96-well plates (subconfluence) or one week in 12-well plates (confluence). Cell media consist of HyClone DMEM (Cytiva, Freiburg in Breisgau, Germany), 1% penicillin/streptomycin, 10% fetal bovine serum, 2.5% HEPES (Pan-Biotech, Aidenbach, Germany), and 1% non-essential amino acids (Pan-Biotech).

Primary porcine RPE cells were prepared from pig eyes as previously described [84]. Eyes were obtained as waste material and byproduct in food production and were used in agreement with the animal welfare officer of the University of Kiel. It is not considered as animal research but rather as an active contribution to limit animal experimentation (German animal welfare act TierSchG). In brief, fat and connective tissues were removed, and the anterior part of the eye cut off. Vitreous body and retina were removed and RPE detached with trypsin followed by a trypsin/EDTA incubation (both Pan-Biotech). Cells were suspended in media and washed two times by centrifugation. Cells were seeded into 12-well or 24-well plates at a density of 100,000 cells per mL (mixed from twelve eyes). Media consist of HyClone DMEM 1% penicillin/streptomycin, 10% fetal bovine serum, 2.5% HEPES, and 1% non-essential amino acids. Cells were cultivated for at least two weeks to reach confluence before experiments were started. For phagocytosis assay cover slips in 12-well plates were coated with collagen I solution (Pan-Biotech) according to manufacturer’s instructions. For measurement of TEER cells were seeded into 12-well plates with transwell membrane inserts (Sarstedt, Nümbrecht, Germany).

All cells were cultivated at 37 °C in a humidified 5% incubator.

### 4.6. Stimulation

Depending on the experiment, cells were treated with 1, 10, 50, or 100 µg/mL fucoidan. Regarding oxidative stress assays OMM-1 were treated with fucoidan for 30 min before stress insult was applied by adding 500 µM H_2_O_2_ (Sigma-Aldrich, St. Louis, MO, USA) or 25 µM erastin (Cayman Chemical, Ann Arbor, MI, USA), and ARPE-19 were treated with fucoidan for 30 min, followed by 250 µM H_2_O_2_ and 20 µM erastin, respectively. Regarding inflammation, cells were treated with 1 µg/mL LPS (Merck, Darmstadt, Germany), 10 µg/mL PIC (Tocris Bioscience, Bristol, UK), 50 ng/mL TNF (R&D System, Minneapolis, MN, USA), or 10 ng/mL Pam2CSK4 (Pam, Tocris Biosciences, Bristol, UK). Depending on the assay, different stimulation times were used as indicated in the result section.

### 4.7. Cell Viability

Cell viability was determined with MTT assay [85]. After washing cells with phosphate-buffered saline (PBS, Pan-Biotech), cells were treated with 0.5 mg/mL MTT (Sigma-Aldrich, St. Louis, MO, USA) for two hours. MTT was removed and cells were dissolved with dimethyl sulfoxide (Carl Roth GmbH + Co. KG, Karlsruhe, Germany). Absorbance was measured with plate reader Elx800 (BioTek, Bad Friedrichshall, Germany) at 550 nm.

### 4.8. Transepithelial Electrical Resistance

Primary porcine RPE were seeded on transwell inserts (refer to Section 4.3). Confluence was controlled by measuring TEER 7, 10, and 14 days after seeding. An empty transwell with media only was used as a blank. After wells reached at least 150 Ohm*cm^2^ confluence was determined [45]. Immediately before treating cells as well as one and three days after treatment TEER was measured to determine changes in barrier function. TEER was measured with Epithelial Volt/Ohm (EVOM™) Meter 3 with STX4 electrodes (WPI, Sarasota, FL, USA). Before each measurement electrodes were washed with 70% ethanol and aqua dest.

### 4.9. Enzyme-Linked Immunosorbent Assay

Secretion of VEGF, IL-6 and IL-8 was measured with ELISA. Supernatants were harvested after indicated stimulation times. In ARPE-19 supernatant was collected for 24 h for VEGF assessment. In primary porcine RPE supernatant was collected for 4 h for VEGF assessment and for 24 h regarding IL-6 and IL-8 testing. Appropriate ELISA DuoSets were purchased from R&D Systems (Minneapolis, MN, USA) and conducted as described in the user manual.

### 4.10. Real-Time Polymerase Chain Reaction

After treatment of primary porcine RPE with SL fucoidans, RNA was isolated with NucleoSpin RNA Kit (Macherey-Nagel, Düren, Germany) as described in the instruction manual. Genomic DNA was removed with DNase from the RNA Kit. RNA was eluted in 20 µL RNase-free water. Concentration and purity of RNA was determined with NanoDrop™ One system (Thermo Fisher Scientific, Waltham, MA, USA). High-Capacity cDNA Reverse Transcription Kit (Thermo Fisher Scientific) was used to generate cDNA as described in the instructions. Real-time PCR was performed with TaqMan™ Fast Advanced Master Mix and TaqMan™ gene expression assays [dye label 5(6)-carboxyfluorescein-minor groove binder (FAM-MGB)] as described in the manual of the master mix. Gene expression of *IL6* (interleukin 6, Ss03384604_u1), *CXCL8* (interleukin 8, Ss03392437_m1), and *GAPDH* (glyceraldehyde-3-phosphate dehydrogenase, Ss03375629_u1) was tested, while the latter was used as endogenous control. The ΔΔCT method was used for evaluation of relative normalized gene expression [86]. ΔCT (=CT [gene of interest]—CT [housekeeping gene]), ΔΔCT (=ΔCT [treated sample]—ΔCT [untreated sample]) and relative fold gene expression level RQ (=2^−ΔΔCT^) were assessed with RQ module of Thermo Fisher Connect.

### 4.11. Western Blotting

Protein expression of RPE65 and CD59 of treated primary porcine RPE cells was assessed with western blot as previously described [27]. Media were removed and cells were washed in PBS buffer. Cell lysates were obtained with Nonidet^®^ P-40 (NP-40, nonylphenyl-polyethylene glycol) lysis buffer, incubated for 45 min on ice. Lysis buffer contained 1% NP-40 substitute (Sigma-Aldrich), 150 mM NaCl (Carl Roth GmbH + Co. KG, Karlsruhe, Germany), 50 mM Tris (Sigma-Aldrich), as well as 1:100 phosphatase inhibitor cocktail I and II and proteinase inhibitor (all from Sigma-Aldrich). Protein concentration was determined with BioRad protein assay as described by the manufacturer (BioRad, Munich, Germany). Bovine serum albumin was used for standard calibration curves. Protein content was measured with Genesys 10 Bio (Thermo Fisher, Waltham, MA, USA) at 595 nm. Proteins were separated with SDS-PAGE in 12% acrylamide gel (15 µg protein amount per sample). The gels were used for western blotting in a wet tank procedure. After transferring proteins, the membranes were blocked with 4% skim milk (Carl Roth GmbH + Co. KG, Karlsruhe, Germany). Membranes were incubated with primary antibodies at 4 °C overnight on a shaker (mouse anti-RPE65 Abcam, 65 kDa, 1:6000, Berlin, Germany; rabbit anti-CD59, 18 kDa, 1:3000, Proteintech Group, Inc., Rosemont, IL, USA; rabbit anti-β-actin, 37 kDa, 1:1000, Cell Signaling Technologies, Denver, CO, USA). Horseradish peroxidase (HRP) conjugates were added for one hour depending on the host of the primary antibodies (anti-mouse-HRP or anti-rabbit-HRP, Cell Signaling Technologies, Denver, CO, USA). As substrate for the HRP, Amersham ECL Western Blotting Detection Reagent (GE Healthcare, Chicago, IL, USA) was used. Chemiluminescent signal was detected with MF-ChemiBIS 1.6 (Biostep, Jahnsdorf, Germany). Band volumes were evaluated with TotalLab TL100 (Biostep). Band volumes of β-actin were used for normalization.

### 4.12. Phagocytosis Assay

For phagocytosis assay confluent RPE cells on collagen I coated cover slips were used (please refer to Section 4.5). After treatment, phagocytosis staining was conducted as previously established [1]. Fluorescence latex beads (Sigma-Aldrich) were opsonized with photoreceptor outer segments prepared from porcine retinae. Opsonized beads were applied to the RPE cells and incubated for four hours. Cells were washed with media and PBS to remove excess beads. Cells were fixated with paraformaldehyde and mounted with Fluoromount-G™ with DAPI (Thermo Fisher Scientific). Fluorescence images were taken with Axiovert Imager M.2 and AxioVision Software (both Zeiss, Jena, Germany). The DAPI stained cell nuclei were counted per hand. Fluorescence beads were counted by self-programmed macro in Fiji (ImageJ2, https://imagej.net/software/fiji/downloads (accessed on 8 August 2022). For evaluation measured beads were set in relation to number of cell nuclei.

### 4.13. Statistical Analysis

Diagrams, means, standard deviation, and visual formatting were made with Microsoft Excel and PowerPoint (Microsoft Office 2010, Microsoft, Redmond, WA, USA). For statistical analysis, GraphPad Prism 9 (GraphPad Software, Inc., San Diego, CA, USA) was used. Normality was checked with the Shapiro–Wilk test. All data of this study showed Gaussian distribution. Relative data were tested with one-sample *t*-test. Absolute data were tested with ANOVA (analysis of variance) followed by post-hoc Dunnett’s multiple comparison test. PCR data were evaluated with Thermo Fisher Connect and integrated student’s *t*-test. Data with *p* values ≤ 0.5 were marked as significant. In general, all significances were calculated between all conditions with the assay control (marked with *) as well as between fucoidans plus proinflammatory agent with proinflammatory agent alone (marked with ^+^).

## 5. Conclusions

The goal of this study was to test two fucoidans from *Saccharina latissima* from Norway (SL-NOR) and the Faroe Islands (SL-FRO) concerning possible beneficial effects against age-related macular degeneration-related pathogenic mechanisms and concerning the maintenance of RPE cell function. It was been previously shown that fucoidans from these species can exert antiangiogenic and antioxidative activities. Primary porcine retinal pigment epithelium was used as well as the human RPE cell line ARPE-19 and the human uveal melanoma cell line OMM-1. We detected no antiproliferative effects in any tested cell model but also no antioxidative effects against erastin or H_2_O_2_ in ARPE-19 and OMM-1. Interleukin 8 gene expression was reduced by SL-NOR. Vascular endothelial growth factor, interleukin 6, and interleukin 8 secretion were reduced by both fucoidans in retinal pigment epithelium. SL-FRO fucoidan additionally reduced vascular endothelial growth factor in ARPE-19. The reduction of CD59 (protectin) expression due to pro-inflammatory stimulus was ameliorated by the fucoidans. Both fucoidans showed complement and elastase inhibitory effects. Retinal pigment epithelium-specific 65 kDa protein was reduced by SL-NOR but not SL-FRO. Barrier function of retinal pigment epithelium by fucoidans was only transient. Phagocytosis ability was not influenced in ARPE-19 (with or without proinflammatory activation). The fucoidans reduced phagocytosis in primary retinal pigment epithelium, which was lost after inflammatory insults. Taken together, two promising fucoidans from *Saccharina latissima* were characterized, which both displayed anti-inflammatory, antiangiogenic, as well as complement inhibitory properties. However, the effect on RPE function and gene expression need to be taken into consideration for further development, which renders SL-FRO to be of higher interest.

## Figures and Tables

**Table 1:** Chemical characteristics of the fucoidans. SL-FRO or SL-NOR were chemically analyzed. *M_w_* = weight average molar mass, *M_n_* = number average molar mass, PD = polydispersity, DS = degree of sulfation, TPC = total phenolic content, and GAE = gallic acid equivalents.

Test Compound	*M_w_* (kDa)	*M_n_* (kDa)	PD (*M_w_*/*M_n_*)	DS	Protein Content (%)	TPC (µg GAE/mg)
SL-FRO	997	238	4.2	0.50	9.7	1.5
SL-NOR	312	190	1.6	0.40	10.7	3.7
Monosaccharide composition (mol%)						
	Fucose	Xylose	Mannose	Galactose	Glucose	Rhamnose
SL-FRO	81.1	5.0	1.8	7.9	1.0	1.0
SL-NOR	73.6	8.2	5.8	9.9	1.2	1.3

**Table 2:** Gene expression of interleukins (three days). Relative gene expression for *IL6* (interleukin 6) and *CXCL8*/*IL8* (interleukin 8) was determined with real-time polymerase chain reaction after treating primary porcine pigment epithelium (RPE) cells with 50 µg/mL SL-FRO or SL-NOR (refer Table 2) with and without 10 µg/mL polyinosinic:polycytidylic acid (PIC) for three days. Relative expression and *p*-values of student’s *t*-test were calculated against control or PIC with Thermo Fisher Connect. co = untreated control, RQ = relative fold gene expression level (=2^−ΔΔCT^), RQ Min = minimal RQ value, RQ Max = maximal RQ values. n = 3 (number of independent experiments).

		vs. Co				vs. PIC			
Bio Group Name	Target Name	RQ	RQ Min	RQ Max	*p*-Value	RQ	RQ Min	RQ Max	*p*-Value
Co	*IL6*	1.000	0.185	5.407	1.000	1.649	0.305	8.916	0.661
PIC	*IL6*	0.606	0.440	0.836	0.661	1.000	0.725	1.379	1.000
SL-FRO	*IL6*	1.055	0.382	2.908	0.965	1.739	0.631	4.796	0.449
SL-FRO+PIC	*IL6*	0.076	0.003	1.802	0.300	0.125	0.005	2.972	0.374
SL-NOR	*IL6*	0.403	0.169	0.963	0.468	0.665	0.278	1.588	0.511
SL-NOR+PIC	*IL6*	0.362	0.120	1.088	0.439	0.597	0.198	1.794	0.507
Co	*IL8*	1.000	0.733	1.365	1.000	0.434	0.318	0.593	0.162
PIC	*IL8*	2.302	1.147	4.619	0.162	1.000	0.498	2.007	1.000
SL-FRO	*IL8*	4.057	0.528	31.153	0.356	1.762	0.230	13.534	0.686
SL-FRO+PIC	*IL8*	11.849	1.616	86.900	0.162	5.147	0.702	37.752	0.288
SL-NOR	*IL8*	0.238	0.136	0.415	0.028	0.103	0.059	0.180	0.013
SL-NOR+PIC	*IL8*	1.896	0.926	3.882	0.260	0.824	0.402	1.687	0.754

**Table 3:** Gene expression of interleukins (seven days). Relative gene expression for IL6 (interleukin 6) and CXCL8 (interleukin 8) was determined with real-time polymerase chain reaction after treating primary porcine pigment epithelium (RPE) cells with 50 µg/mL SL-FRO or SL-NOR (refer Table 2) with and without 10 µg/mL polyinosinic:polycytidylic acid (PIC) for seven days. Relative expression and *p*-values of student’s *t*-test were calculated against control or PIC with Thermo Fisher Connect. co = untreated control, RQ = relative fold gene expression level (=2^−ΔΔCT^), RQ Min = minimal RQ value, RQ Max = maximal RQ values. n = 3 (number of independent experiments).

		vs. Co				vs. PIC			
Bio Group Name	Target Name	RQ	RQ Min	RQ Max	*p*-Value	RQ	RQ Min	RQ Max	*p*-Value
Co	*IL6*	1.000	0.224	4.473	1.000	0.570	0.127	2.550	0.586
PIC	*IL6*	1.754	1.216	2.529	0.586	1.000	0.694	1.442	1.000
SL-FRO	*IL6*	2.146	1.575	2.925	0.472	1.224	0.898	1.668	0.507
SL-FRO+PIC	*IL6*	4.130	2.702	6.314	0.238	2.355	1.541	3.600	0.058
SL-NOR	*IL6*	3.424	2.588	4.531	0.289	1.953	1.476	2.584	0.070
SL-NOR+PIC	*IL6*	4.930	2.110	11.523	0.202	2.811	1.203	6.57	0.158
Co	*IL8*	1.000	0.897	1.115	1.000	0.270	0.242	0.301	0.134
PIC	*IL8*	3.704	1.456	9.422	0.134	1.000	0.393	2.544	1.000
SL-FRO	*IL8*	50.009	27.133	92.175	0.007	13.500	7.324	24.882	0.021
SL-FRO+PIC	*IL8*	120.378	50.660	286.040	0.010	32.496	13.676	77.216	0.009
SL-NOR	*IL8*	1.002	0.468	2.142	0.997	0.270	0.126	0.578	0.136
SL-NOR+PIC	*IL8*	1.391	0.236	8.217	0.778	0.376	0.064	2.218	0.459

**Table 4:** Algal species, their harvest dates and sites.

Fucoidan	Algae Species	Harvest Date	Origin
SL-FRO	*Saccharina latissima*	July 2016	Coast of Faroe Islands
SL-NOR	*Saccharina latissima*	May 2017	Coast of Norway

## Data Availability

Data are available on request.
